# Aberrant CDK4/6-driven cell-cycle reentry drives neuronal loss and defines a therapeutic target in C9orf72 ALS/FTD

**DOI:** 10.1016/j.isci.2025.114596

**Published:** 2026-01-02

**Authors:** Ling Lian, Hayley Robinson, Noah Daniels, G. Aleph Prieto, Gunnar H.D. Poplawski, Rodrigo Lopez-Gonzalez

**Affiliations:** 1 Department of Neurosciences, Lerner Research Institute, Cleveland Clinic, Cleveland, OH 44195, USA; 2 Instituto de Neurobiologia, Universidad Nacional Autonoma de Mexico, Queretaro, Mexico; 3 Center for Immunotherapy and Precision Immuno-Oncology, Lerner Research Institute, Cleveland Clinic, Cleveland, OH 44195, USA

**Keywords:** Health sciences, Pharmaceutical science, Neuroscience, Clinical neuroscience, Cellular neuroscience

## Abstract

The C9orf72 hexanucleotide repeat expansion (G4C2) is the most common genetic cause of amyotrophic lateral sclerosis (ALS) and frontotemporal dementia (FTD), yet targeted therapies remain unavailable. Here, we show that induced pluripotent stem cell (iPSC)-derived post-mitotic neurons from C9orf72 carriers exhibit age-dependent cell-cycle reentry, increased S-phase entry, and elevated cyclin and CDK expression. Mechanistically, arginine-containing dipeptide repeat proteins (poly-GR and poly-PR) translated from G4C2 repeats drive this aberrant activation through stimulation of the CDK4/6 pathway, whereas poly-GP and C9orf72 loss-of-function show no effect. Importantly, the FDA-approved CDK4/6 inhibitor palbociclib normalizes cell-cycle progression, reduces S-phase entry, decreases motor neuron death, and restores synaptic proteins PSD95 and synapsin-1. Single-nucleus RNA sequencing from C9orf72 patient cortex reveals cell-cycle activation within excitatory neuron subclusters and alterations in DNA repair and cell-cycle regulation pathways, supporting our *in vitro* findings. These findings establish cell-cycle dysregulation as a central pathogenic mechanism in C9orf72 ALS/FTD and highlight CDK4/6 signaling as a promising therapeutic target.

## Highlights

• C9orf72 neurons show age-dependent cell-cycle reentry and S-phase activation
• Arginine-rich DPRs (poly-GR/PR) drive CDK4/6-mediated cell-cycle dysregulation
• CDK4/6 inhibition with palbociclib restores neuronal survival and synaptic markers
• snRNA-seq of C9orf72 cortex reveals excitatory neuron-specific cell-cycle activation

## Graphical abstract

**Figure:**
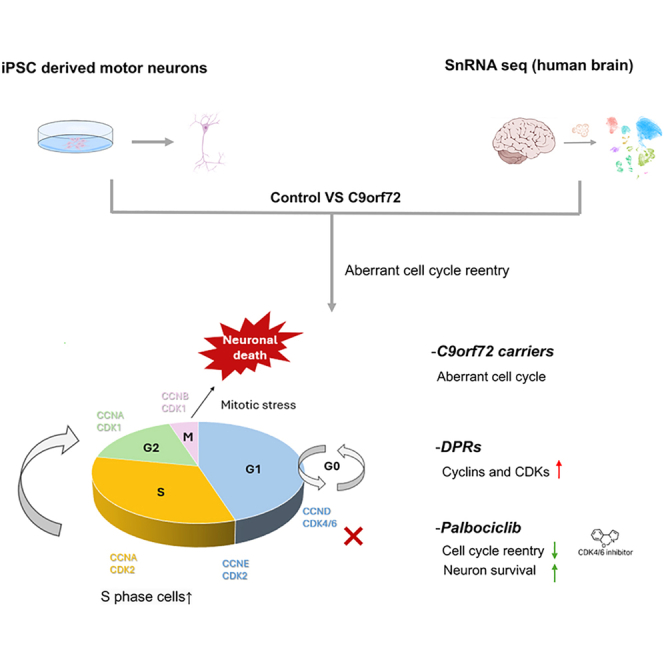

## Introduction

Amyotrophic lateral sclerosis (ALS) and frontotemporal dementia (FTD) are fatal neurodegenerative diseases for which there are currently no effective treatments. The GGGGCC (G4C2) repeat expansion in the *C9orf72* gene is the most common genetic cause of familial ALS and FTD.^1^^,^^2^ Three non-mutually exclusive mechanisms are implicated in *C9orf72* pathogenesis: (1) haploinsufficiency of the *C9orf72* protein; (2) formation of RNA foci from bidirectionally transcribed repeat RNA; and (3) repeat-associated non-AUG (RAN) translation of dipeptide repeat proteins (DPRs): GA, GR, PR, PA, and GP.^3^^,^^4^^,^^5^ Recent studies have identified genome instability as a central driver of neurodegeneration in *C9orf72* repeat expansion.^6^^,^^7^^,^^8^^,^^9^^,^^10^ We previously demonstrated that arginine-containing DPRs cause DNA damage, impair DNA repair, and that p53 knockdown can rescue neuronal viability both *in vitro* and *in vivo*,^11^^,^^12^ suggesting that modulation of the DNA damage response could offer therapeutic benefit.

Neurons are terminally differentiated and normally exist in a quiescent G0 state. DNA damage can induce aberrant cell-cycle reentry in post-mitotic neurons, ultimately triggering apoptosis.^13^^,^^14^^,^^15^ The cell-cycle is governed by precise interactions between cyclins and cyclin-dependent kinases (CDKs). In particular, the Cyclin D/CDK4/6 complex facilitates G1/S transition by phosphorylating retinoblastoma protein (RB), thereby releasing the transcriptional factor E2F that initiate S-phase entry.^16^^,^^17^^,^^18^ Dysregulation of this machinery has been observed in ALS patient tissue, including increased phosphorylated RB, E2F1, p53, p16, and p21 levels in spinal cord and cortical neurons.^19^^,^^20^^,^^21^

In this study, we investigated whether cell-cycle dysregulation is a pathological feature of *C9orf72*-associated ALS/FTD. Using human induced pluripotent stem cell (iPSC)-derived post-mitotic motor neurons from *C9orf72* repeat expansion carriers, we observed age-dependent increases in S-phase re-entry, cyclins, CDKs, and cell division markers compared to controls. We show that treatment with the CDK4/6 inhibitor palbociclib significantly reduces S-phase entry and enhances neuronal survival. Finally, the analysis of single-nucleus RNA sequencing (snRNA-seq) datasets from postmortem *C9orf72* patient brains revealed cell-cycle abnormalities in excitatory neurons, including elevated G1/S and S-phase scores and evidence of copy number variation (CNV) in regions enriched for DNA repair and cell-cycle regulatory genes. Collectively, these findings identify aberrant cell-cycle activation as a disease mechanism in *C9orf72*-ALS/FTD and support CDK4/6 inhibition as a potential therapeutic strategy.

## Results

### C9orf72 neurons show age-dependent cell-cycle reentry

**Figure 1:**
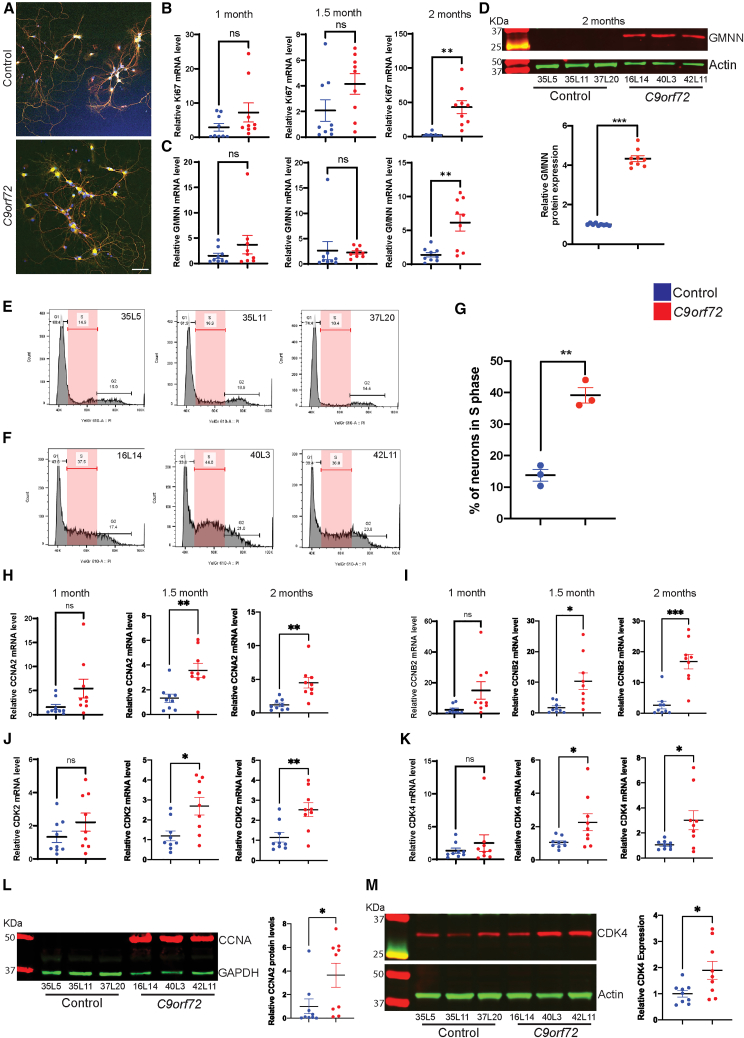
Post-mitotic iPSC-derived motor neurons from C9orf72 carriers aberrantly enter cell-cycle (A) Representative images of control and *C9orf72* motor neuron cultures. Scale bars, 100 μm. (B–C) mRNA levels of Ki67 and GMNN at 1-, 1.5-, and 2-month-old control and *C9orf72* iPSC-derived motor neurons. (D) Representative western blot images of GMNN and actin and quantification of protein levels from 2-month-old control and *C9orf72* iPSC-derived motor neurons. (E–F) Flow cytometry of propidium iodide-stained 2-month-old iPSC-derived motor neurons from control (lines: 35L5, 35L11, and 37L20) and *C9orf72* (lines: 16L14, 40L3, and 42L11). (G) Percentage of neurons in S-phase from controls and *C9orf72* neurons. (H and I) mRNA levels of CCNA2 and CCNB2 at 1-, 1.5-, and 2-month-old control and *C9orf72* iPSC-derived motor neurons. (J and K) mRNA levels of CDK2 and CDK4 at 1-, 1.5-, and 2-month-old control and *C9orf72* iPSC-derived motor neurons. (L) Representative western blot images of CCNA2 and GAPDH and quantification of protein levels from 2-month-old control and *C9orf72* iPSC-derived motor neurons. (M) Representative western blot images of CDK4 and actin and quantification of protein levels from 2-month-old control and *C9orf72* iPSC-derived motor neurons. Data are presented as mean ± SEM (B–D, G–M). Data presented in (B–D) is from 3 control and 3 *C9orf72* iPSC iPSC-derived neuron cultures from 3 independent differentiation experiments. Two-tailed *t* test with Welch’s correction was applied. ns, not significant, ∗*p* < 0.05, ∗∗*p* < 0.01, and ∗∗∗*p* < 0.001. Data presented in (G) is from 3 control and 3 *C9orf72* iPSC-derived neuron cultures. Two-tailed *t* test with Welch’s correction was applied. ns, not significant, ∗*p* < 0.05. Data presented in (H–K) 3 control and 3 *C9orf72* iPSC-derived neuron cultures from 3 independent differentiation experiments. Two-tailed *t* test with Welch’s correction was applied. ns, not significant, ∗*p* < 0.05 and ∗∗*p* < 0.01. Data presented in (L and M) is from 3 control and 3 *C9orf72* iPSC-derived neuron cultures from 3 independent differentiation experiments. Two-tailed *t* test with Welch’s correction was applied. ∗*p* < 0.05. In cases where the loading control was located above the target protein on the original membrane, the band was repositioned below for consistency and clarity.

We differentiated motor neurons from three control iPSC lines and three *C9orf72* lines (see “methods)” using established protocols.^6^^,^^11^ Cultures consisted of >90% post-mitotic motor neurons (Figure 1A). We measured Ki67 and Geminin (GMNN) mRNA as cell division markers. At 1 and 1.5 months the levels were comparable, but at 2 months both Ki67 and GMNN transcripts were significantly higher in *C9orf72* neurons than controls (Figures 1B and 1C). To confirm pathway activation, we measured protein levels of GMNN, and we found a significant increase in *C9orf72* neurons at 2 months in agreement with increases in mRNA levels. (Figure 1D). To directly measure cell-cycle progression, we performed flow cytometry on propidium iodide-stained neurons. The fraction of cells in S-phase was significantly greater in 2-month-old *C9orf72* cultures than in controls (Figures 1E–1G). Thus, more *C9orf72* neurons re-enter the cell-cycle. Next, we examined cyclin and CDK expression. Relative to controls, *C9orf72* neurons showed significantly increased mRNA for Cyclin A2 (CCNA2) and Cyclin B2 (CCNB2) at 1.5 and 2 months (Figures 1H and 1I). Similarly, transcripts for CDK2 and CDK4 were elevated in 1.5- and 2-month *C9orf72* neurons (Figures 1J and 1K). In contrast, levels of other cyclins (CCNC, CCND1, CCND2, CCNE1, and CCNE2) and CDK1 did not differ significantly between groups (Figure S1). Immunoblot analysis confirmed higher protein levels of CCNA2 and CDK4 in 2-month *C9orf72* neurons (Figures 1L–1M). These data demonstrate that *C9orf72* neurons progressively upregulate cell-cycle machinery and enter S-phase.

### Toxic DPRs, not C9orf72 deficiency, activate cell-cycle machinery in motor neurons

**Figure 2:**
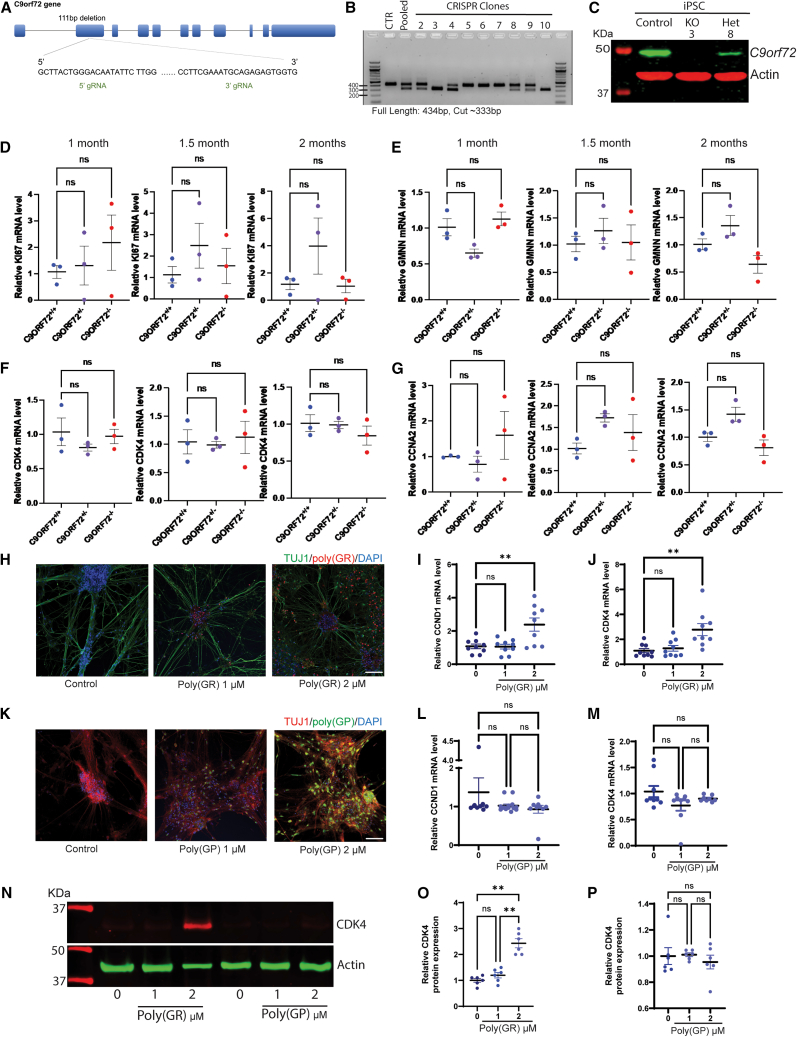
Poly (GR) induces an increase in cyclins and CDKs levels (A) Schematic representation of the CRISPR/Cas9 strategy to generate *C9orf72* homozygous and heterozygous lines from a healthy control line. (B) Generation of *C9orf72* heterozygous and homozygous knockout iPSC lines by CRISPR Cas9. (C) Representative western blot image of *C9orf72* protein levels in homozygous (line 3) and heterozygous (line 8) knockout lines by CRISPR Cas9. (D–G) mRNA levels of Ki67, GMNN, CDK4, and CCNA2 at 1-, 1.5-, and 2-month-old control and *C9orf72* iPSC-derived motor neurons. (H) Representative immunostaining images of iPSC-derived motor neurons cultures treated with 1 and 2 μM of poly (GR). Scale bars, 200 μm. (I and J) Quantification of protein levels of CCND1 and CDK4 in 2-month-old control iPSC-derived motor neurons treated with 1 and 2 μM of poly (GR). (K) Representative immunostaining images of iPSC-derived motor neurons cultures treated with 1 and 2 μM of poly (GP). Scale bars, 200 μm. (L and M) Quantification of protein levels of CCND1 and CDK4 in 2-month-old control iPSC-derived motor neurons treated with 1 and 2 μM of poly (GR). (N) Representative western blot image of control iPSC-derived neurons treated with Poly (GR) and poly (GP). (O and P) Quantification of protein levels of CDK4 in 2-month-old control iPSC-derived motor neurons treated with 1 and 2 μM of poly (GR) and poly (GP). Data are presented as mean ± SEM (D–G, I and J, L and M, O and P). Data presented in (D–G) is from 3 independent differentiation experiments of a control iPSC line (parental line) and one *C9orf72* heterozygous and one homozygous knockout line. Two-tailed *t* test with Welch’s correction was applied. ns, not significant. Data in (I and J, L and M) is from 3 control iPSC lines treated with DPRs from 3 independent differentiation experiments. Two-tailed *t* test with Welch’s correction was applied. ns, not significant, ∗∗*p* < 0.01. Data in (O and P) is from 3 control iPSC lines treated with DPRs from 2 independent differentiation experiments. Two-tailed *t* test with Welch’s correction was applied. ns, not significant, ∗∗*p* < 0.01. In cases where the loading control was located above the target protein on the original membrane, the band was repositioned below for consistency and clarity.

To dissect the molecular mechanisms underlying cell-cycle dysregulation in *C9orf72* neurons, we first examined whether *C9orf72* haploinsufficiency contributes to this phenotype. We generated heterozygous *C9orf72*^+/−^ and homozygous *C9orf72*^−/−^ knockout lines from a control iPSC line *C9orf72*^+/+^ using CRISPR/Cas9 (Figures 2A–2C). Following characterization, we selected one heterozygous (line 8) and one homozygous (line 3) knockout line for motor neuron differentiation. Analysis of cell-cycle markers in these neurons at 1, 1.5, and 2 months revealed no significant differences in Ki67, GMNN, CCNA2, CDK4, CCNB1, CCNB2, or CDK1 mRNA levels compared to parental *C9orf72*^+/+^ motor neurons (Figures 2D–2G and S2A–S2C). These results indicate that *C9orf72* haploinsufficiency alone does not drive cell-cycle dysregulation in motor neurons.

We next investigated the role of dipeptide repeat proteins (DPRs), which are generated through RAN translation of the G4C2 expansion. Among the five DPR species produced, the arginine-containing glycine-arginine (GR) and proline-arginine (PR) peptides exhibit the highest neurotoxicity.^6^ To assess their impact on cell-cycle regulation, we treated 1-month-old control iPSC-derived motor neurons from three independent lines with synthetic peptides containing 20 repeats of GR or PR (GR_20_ and PR_20_) treatment with GR_20_ significantly increased mRNA expression of both CCND1 and CDK4 (Figures 2H–2J) and CDK4 protein levels (Figures 2N–2O). Similarly, PR_20_ treatment elevated CDK4 transcript levels (Figure S2D). In contrast, GR_20_ did not alter expression of GMNN, Ki67, CCNA2, or CCNB2 (Figures S2E–S2K). To determine if the increase in cell-cycle activation is specific to arginine-containing DPRs we treated cells with poly(GP). Poly(GP) treatment did not significantly increase CCND1 and CDK4 mRNA levels (Figures 2K–2M) and the protein levels of CDK4 (Figures 2N and 2P). These findings demonstrate that arginine-containing DPRs selectively upregulate specific G1/S-phase regulators, implicating them as key drivers of cell-cycle dysregulation in *C9orf72* disease.

### CDK4/6 inhibition suppresses cell-cycle reentry in C9orf72 motor neurons

**Figure 3:**
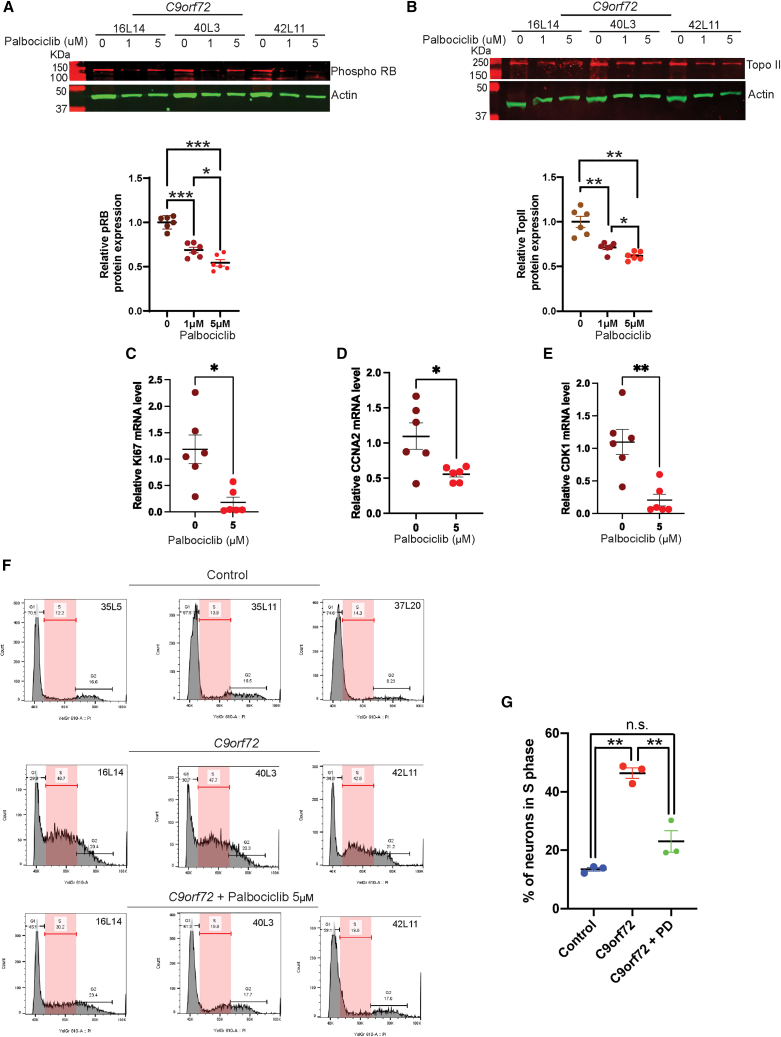
CDK4/6 inhibitor Palbociclib (PD33002291) prevents cell-cycle reentry in iPSC-derived motor neuron from *C9orf72* carriers (A and B) Western blot and quantification of protein levels of phosphorylated RB and topoisomerase II α (TopoII) in 2-month-old iPSC-derived motor neurons from *C9orf72* carriers treated with Palbociclib 1 and 5 μM. (C–E) mRNA levels of Ki67 and CCNA2 and CDK1 in 2-month-old *C9orf72* iPSC-derived motor neurons treated with Palbociclib 5 μM. (F) Flow cytometry of propidium iodide-stained 2-month-old iPSC-derived motor neurons from controls, *C9orf72* and *C9orf72* neurons treated with Palbociclib 5 μM. (G) Quantification of the percentage of *C9orf72* iPSC-derived motor neurons in S-phase. Data are presented as mean ± SEM (A–E, G). Data presented in (A and B) is from iPSC-derived neurons from 3 *C9orf72* and 3 *C9orf72* treated with Palbociclib 1 and 5 μM from 2 differentiation experiments, one-way ANOVA with Newman-Keuls post hoc test was applied ∗*p* < 0.05.∗∗*p* < 0.01 and ∗∗∗*p* < 0.001. Data presented in (C–E) is from iPSC-derived neurons from 3 *C9orf72* and 3 *C9orf72* neurons with Palbociclib 5 μM from 2 differentiation experiments. Two-tailed *t* test with Welch’s correction was applied ∗*p* < 0.05 and ∗∗*p* < 0.01. Data in (G) is from iPSC-derived neurons from 3 controls, 3 *C9orf72*, and 3 *C9orf72* treated with Palbociclib from 1 differentiation experiment, one-way ANOVA with Newman-Keuls post hoc test was applied ∗∗*p* < 0.01. See also Figure S3.

To test if blocking G1/S entry could prevent neuronal cell-cycle reentry, we treated 1-month-old *C9orf72* motor neuron cultures with the CDK4/6 inhibitor palbociclib (1 or 5 μM) for 1 month. Palbociclib effectively decreased RB phosphorylation as expected (Figure 3A). Consistent with reduced E2F activity, protein levels of topoisomerase IIα, an E2F target, were also reduced (Figure 3B). Quantitative PCR showed that palbociclib treatment significantly lowered Ki67, CCNA2, and CDK4 mRNA in *C9orf72* neurons (Figures 3C–3E). Importantly, flow cytometry revealed that palbociclib (5 μM) markedly reduced the percentage of *C9orf72* neurons in S-phase (Figures 3F and 3G). Thus, chronic CDK4/6 inhibition prevents aberrant cell-cycle progression in these neurons.

### CDK4/6 inhibition rescues neuronal survival and synaptic functionality in C9orf72 motor neurons

**Figure 4:**
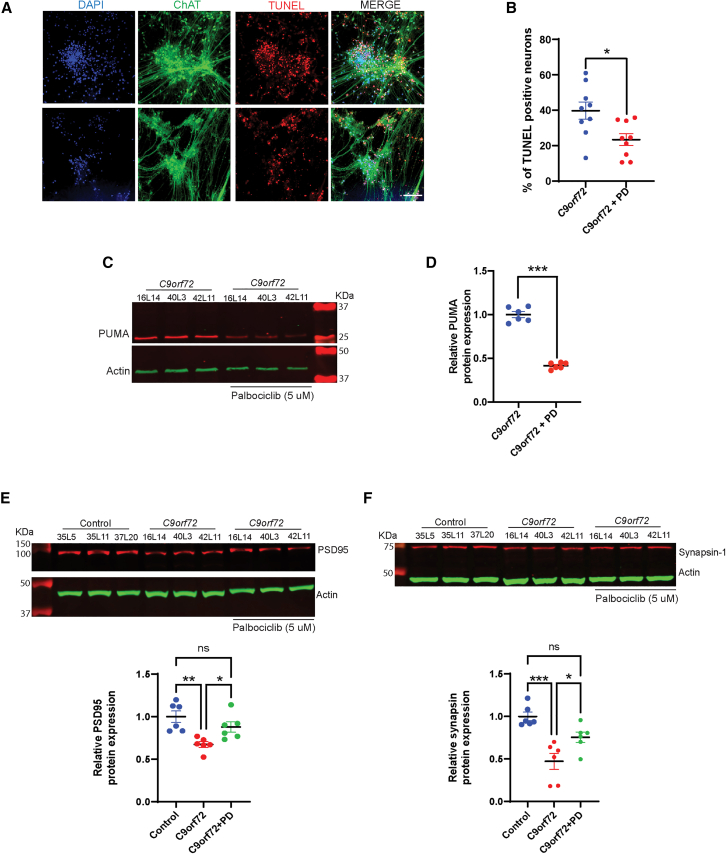
Palbociclib rescues neuronal survival, suppresses pro-apoptotic signaling, and restores synaptic integrity in *C9orf72* neurons (A) Representative images of ChAT and TUNEL positive iPSC-derived motor neurons treated with Palbociclib 5 μM. Scale bars, 150 μm. (B) Quantification of immunostained ChAT and TUNEL positive cells in 2-month-old iPSC-derived motor neurons from *C9orf72* carriers and *C9orf72* carriers treated with Palbociclib 5 μM. (C) Representative western blots of PUMA in iPSC-derived neurons from iPSC-derived neurons from *C9orf72* and *C9orf72* treated with Palbociclib 5 μM. (D) Western blot quantification of PUMA levels iPSC-derived neurons from *C9orf72* and *C9orf72* treated with Palbociclib 5 μM. (E and F) Representative western blots showing PSD95 and Synapsin-1 levels in control and C9orf72 in iPSC-derived neurons with or without Palbociclib treatment and western blot quantification. Data are presented as mean ± SEM (B, D–F). Data in (A and B) is from iPSC-derived motor neurons from 3 *C9orf72* and 3 *C9orf72* treated with Palbociclib from 3 independent differentiation experiments Two-tailed *t* test with Welch’s correction was applied, ∗*p* < 0.05. Data in (C and D) is from iPSC-derived motor neurons from 3 *C9orf72* and 3 *C9orf72* treated with Palbociclib from 2 independent differentiation experiments two-tailed *t* test with Welch’s correction was applied, ∗∗∗*p* < 0.001. Data in (E and F) is from iPSC-derived motor neurons, 3 controls, 3 *C9orf72*, and 3 *C9orf72* treated with Palbociclib from 2 independent differentiation experiments two-tailed *t* test with Welch’s correction was applied, ∗*p* < 0.05, ∗∗*p* < 0.01, and ∗∗∗*p* < 0.001. In cases where the loading control was located above the target protein on the original membrane, the band was repositioned below for consistency and clarity.

To evaluate whether CDK4/6 inhibition promotes neuronal survival, *C9orf72* motor neuron cultures were maintained for one month in 5 μM palbociclib. This treatment significantly reduced the proportion of TUNEL-positive ChAT^+^ motor neurons (Figures 4A and 4B) and decreased protein levels of the pro-apoptotic marker PUMA (Figures 4C and 4D), indicating enhanced neuronal viability. To further assess neuronal health and functionality, the synaptic markers PSD95 and Synapsin-1 were quantified by western blot. *C9orf72* neurons exhibited significantly reduced levels of both proteins compared with controls, consistent with synaptic impairment. Notably, palbociclib treatment restored PSD95 and Synapsin-1 expression to near-control levels (Figures 4E and 4F), suggesting that CDK4/6 inhibition not only prevents aberrant cell-cycle reentry and apoptosis but also promotes synaptic integrity and functional recovery in *C9orf72* motor neurons.

### Single-nucleus RNA-seq reveals aberrant cell-cycle activation in C9orf72 patient neurons

**Figure 5:**
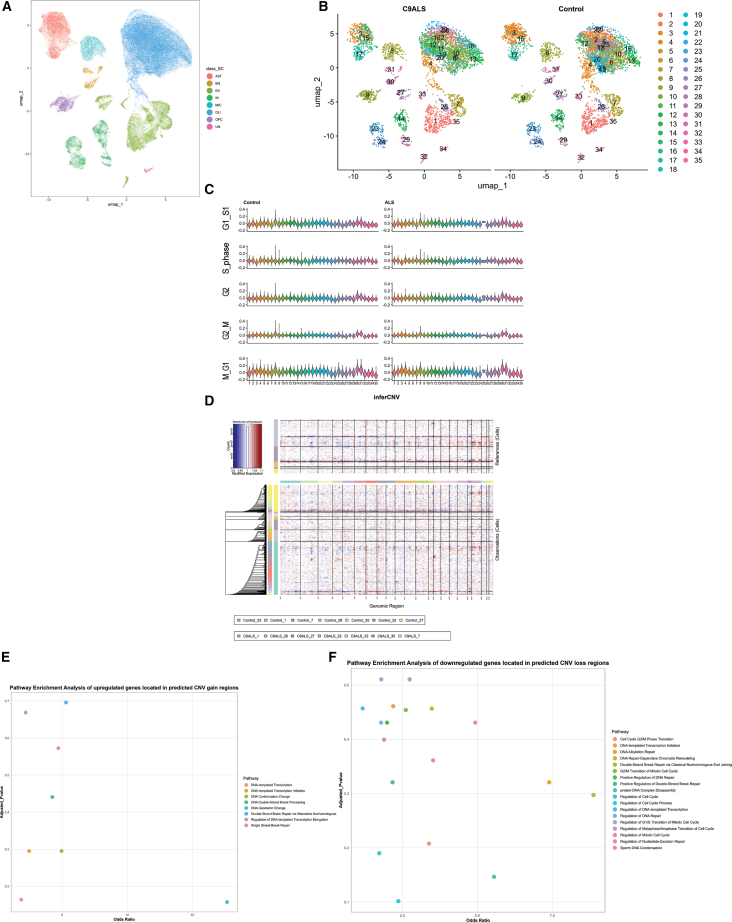
snRNA-seq analysis revealed cell-cycle alterations in excitatory neurons from *C9orf72/*ALS repeat expansion carriers (A) UMAP plot of single-nucleus RNA sequencing data from control and ALS groups. Each dot represents a single mononuclear cell, and colors indicate different cell types: AST (red), EN (brown), EX (yellow), IN (green), MIC (cyan), OLI (blue), OPC (green), and UN (pink). (B) UMAP plot of comparison of cell-type distribution between control and ALS groups. In total, 35 subclusters are annotated. 1, 7, 26, 27, 32, 33, and 35 are excitatory neuron subclusters. (C) Violin plots showing the distribution of the cell-cycle phase scores in all subclusters between control and ALS groups. (D) Heatmap of estimation of copy number variants among all excitatory neurons via the InferCNV algorithm, indicating CNV trend at different chromosome locations(*x* axis). Red dots represent predicted CNV gain regions, and blue dots represent predicted CNV loss regions. (E and F) Scatterplot illustrates pathways related to cell-cycle events enriched from genes located in the predicted CNV gain regions via GO enrichment analysis.

To validate our *in vitro* findings in human brain tissue, we analyzed single-nucleus RNA sequencing (snRNA-seq) data from postmortem cortical samples of *C9orf72* ALS patients and age-matched controls. Using Uniform Manifold Approximation and Projection (UMAP), we identified major cell types including excitatory neurons (EX), inhibitory neurons (IN), microglia (MIC), astrocytes (AST), endothelial cells (EN), oligodendrocytes (OLI), oligodendrocyte precursor cells (OPC), and unclassified cells (UN) (Figure 5A). Comparison of cellular landscapes between control and ALS samples revealed striking differences in cell type distribution and clustering patterns (Figure 5B). Control samples displayed compact, well-organized cell clusters characteristic of healthy brain architecture. In contrast, ALS samples showed altered cellular distributions with notable changes in microglial (subcluster 8) and astrocytic (subclusters 3, 15, and 17) populations, accompanied by reduced excitatory neuron density. The excitatory neuron population was particularly affected, with multiple subclusters (1, 7, 26, 27, 32, 33, and 35) showing dispersed distributions and reduced cellular density, suggesting neuronal loss and dysfunction (Figure S4A). To assess cell-cycle dysregulation, we calculated phase-specific scores for G1/S, S, G2, G2/M, and M phases using Seurat’s AddModuleScore function with established cell-cycle gene sets.^22^ Control excitatory neurons exhibited scores centered around zero across all phases, confirming their expected quiescent G0 state (Figure 5C). Strikingly, ALS excitatory neurons displayed significantly elevated and broadly distributed cell-cycle scores, indicating aberrant cell-cycle reentry in post-mitotic neurons. Subcluster analysis revealed heterogeneous responses to disease. Subcluster 35 showed the most pronounced alterations, with markedly elevated G1/S- and S-phase scores in ALS samples, while subcluster 32 demonstrated increased scores across all cell-cycle phases. Conversely, subclusters 1, 7, and 27 showed minimal differences between ALS and controls, suggesting cell type-specific vulnerability to cycle dysregulation. Statistical analysis confirmed that S-phase alterations were the most significant across excitatory neuron subclusters, corroborating our *in vitro* findings of increased S-phase entry in *C9orf72* neurons (Figure S4B). As expected, proliferative cell types including microglia (subcluster 8) and endothelial cells (subclusters 30 and 31) maintained elevated cell-cycle scores in both control and ALS samples, validating our analytical approach.

### Copy number and pathway analysis implicates genome instability

We next examined genomic instability in these neurons via inferred CNV. InferCNV analysis of excitatory neurons revealed predicted CNV gain (red) and loss (blue) regions across chromosomes (Figure 5D). We extracted genes from the gain or loss regions and performed gene ontology (GO) enrichment. Genes in the gain regions were enriched for DNA-templated transcription, DNA conformation change, double-strand break processing, single-strand break repair, and alternative non-homologous end joining (NHEJ) repair (Figure 5E). Genes in loss regions were enriched for cell-cycle regulation (mitotic G1/S and G2/M transitions) and various DNA repair processes (chromatin remodeling, double-strand break repair, etc.) (Figure 5F). These findings suggest that excitatory neurons in *C9orf72* brains undergo genomic changes affecting DNA repair and cell-cycle pathways.

## Discussion

Our study establishes aberrant cell-cycle reentry as a targetable pathogenic mechanism in C9orf72 ALS/FTD, this study provides proof-of-concept that CDK4/6 inhibition can normalize aberrant neuronal cell-cycle reentry in C9orf72 ALS/FTD. We demonstrate that C9orf72 neurons undergo age-dependent cell-cycle dysregulation characterized by increased S-phase entry, elevated cyclin/CDK expression, and neuronal death, all of which can be rescued by the FDA-approved CDK4/6 inhibitor palbociclib.

Our mechanistic studies reveal that arginine-containing dipeptide repeat proteins (poly-GR and poly-PR), rather than C9orf72 haploinsufficiency, drive pathological cell-cycle activation. This finding is particularly significant given that poly-GR expression correlates with neurodegeneration in patient tissues^23^ and induces DNA damage and genome instability.^24^ The selective upregulation of G1/S regulators (CCND1 and CDK4) by these toxic DPRs provides a direct molecular link between C9orf72 pathology and cell-cycle dysfunction. These observations align with emerging evidence that DNA damage-induced cell-cycle reentry represents a convergent mechanism in neurodegeneration. Previous studies have documented elevated cell-cycle markers (p16, p21, phospho-RB, and E2F1) in ALS patient spinal cord and cortex,^19^^,^^20^^,^^21^ while G4C2 repeat expression disrupts cell-cycle and DNA repair protein distribution in neurons.^25^ Recent work shows that *C9orf72* loss disrupts primary cilia structure and signaling that normally maintain neuronal quiescence. Because cilia help restrain cell-cycle entry, their dysfunction may further predispose *C9orf72* neurons to inappropriate reactivation of the cell-cycle machinery. Thus, cilia deficits from *C9orf72* loss-of-function may act alongside DPR toxicity to promote aberrant cell-cycle reentry.^26^ Our findings extend this framework by identifying the specific molecular drivers and demonstrating their therapeutic accessibility.

The efficacy of palbociclib in our human C9orf72 neuronal models represents a significant therapeutic advance. Chronic CDK4/6 inhibition not only prevented aberrant S-phase entry through expected mechanisms, reducing RB phosphorylation and E2F activity, but also rescued neuronal survival with a 42% reduction in motor neuron death and a decrease in PUMA levels. This dual effect of normalizing cell-cycle progression while enhancing neuronal viability, positions CDK4/6 inhibitors as promising candidates for clinical translation. Importantly, the restoration of PSD95 and Synapsin-1 levels following palbociclib treatment supports the conclusion that CDK4/6 inhibition rescues overall neuronal function rather than merely preventing cell death. Synaptic loss is an early and progressive feature of ALS/FTD pathology,^27^^,^^28^ and normalization of these synaptic proteins suggests a functional recovery of neuronal connectivity. Together with reduced TUNEL reactivity, these findings indicate that cell-cycle normalization by palbociclib preserves both viability and synaptic integrity in C9orf72 neurons, strengthening its potential as a disease-modifying approach. Our findings complement other successful approaches targeting DNA damage response pathways in C9orf72 models, including partial Ku80 inhibition^11^ and PARP inhibition for TDP-43 toxicity.^29^ However, palbociclib offers distinct advantages: FDA approval with established safety profiles, blood-brain barrier penetration, and direct targeting of the aberrant cell-cycle machinery we identify as central to C9orf72 pathogenesis.

Single-nucleus RNA sequencing analysis of C9orf72 patient cortex provides crucial validation of our *in vitro* findings. The identification of excitatory neuron subclusters with elevated G1/S and S-phase scores confirms that post-mitotic neurons inappropriately re-enter the cell-cycle in human disease. The heterogeneous vulnerability across neuronal subclusters (with subclusters 32 and 35 most severely affected) may explain selective neuronal loss patterns in C9orf72 ALS/FTD. Furthermore, our CNV analysis reveals genomic instability affecting DNA repair and cell-cycle regulatory pathways, providing a potential mechanistic link between DNA damage and cell-cycle dysregulation. The enrichment of alternative NHEJ repair genes in CNV gain regions and cell-cycle transition genes in loss regions suggests a complex interplay between genome maintenance and cell-cycle control that warrants further investigation.

Overall, our work establishes cell-cycle dysregulation as a central, druggable mechanism in C9orf72 ALS/FTD. By demonstrating that arginine-containing DPRs drive CDK4/6 pathway activation, and that pharmacological inhibition rescues neuronal survival and synaptic function. Future studies should explore combination therapies targeting both cell-cycle and DNA repair pathways, investigate the long-term effects of CDK4/6 inhibition in animal models, and determine optimal therapeutic windows for intervention. Additionally, the heterogeneous neuronal vulnerability we observe suggests that precision approaches targeting specific neuronal subtypes may maximize therapeutic benefit.

### Limitations of the study

The conclusions of our study are based on iPSC-derived human motor neurons, which, while valuable for modeling cell-autonomous phenotypes, lack the broader circuit-level and glial interactions that may influence cell-cycle dysregulation *in vivo*. Although CDK4/6 inhibition reduced aberrant cell-cycle activation and restored synaptic markers, the downstream pathways linking CDK4/6 activity to neuronal survival remain to be defined. Additionally, the snRNA-seq analyses identify transcriptional signatures consistent with cell-cycle reentry, but do not resolve whether these alterations originate from specific neuronal subtypes or reflect convergent stress-response pathways. Further *in vivo* and multi-omic approaches will be required to delineate the precise mechanisms through which *C9orf72* pathology engages the cell-cycle machinery to drive neurodegeneration.

## Resource availability

### Lead contact

Further information and requests for resources and reagents should be directed to and will be fulfilled by the lead contact, Rodrigo Lopez-Gonzalez (lopezgr@ccf.org).

### Materials availability

All the materials generated from this manuscript will be available under reasonable request.

### Data and code availability

• All data used for single-nucleus RNA sequencing analysis in this study are available from the GEO database under accession number GSE219281.
• The custom code used for data analysis has been deposited on Zenodo and is publicly available at: https://doi.org/10.5281/zenodo.15593963.
• Any additional information required to reanalyze the data reported in this paper is available from the lead contact upon request.

## Acknowledgments

We thank the Cleveland Clinic for assistance with flow cytometry training and experiments. This work was partially funded by 10.13039/100000065NINDS 1K01NS136791-01A1 grant awarded to R.L.G.

## Author contributions

R.L.G. conceived, supervised the project, and wrote the manuscript with input from G.P. Most of the experiments were conducted by L.L. with help from H.R. and N.D. Flow cytometry experiment analysis was performed by A.P.

## Declaration of interests

The authors declare no conflicts of interests or competing financial interests.

## STAR★Methods

### Key resources table

**Table:** 

REAGENT or RESOURCE	SOURCE	IDENTIFIER
**Antibodies**		

Rabbit anti-Geminin (GMNN)	Proteintech	Cat# 10802-1-AP; RRID:AB_2110945
Rabbit anti-Cyclin A2 (CCNA2)	Cell Signaling	Cat# 67955; RRID:AB_2909603
Rabbit anti-CDK4	Cell Signaling	Cat# 12790; RRID:AB_2631166
Rabbit anti-Phospho-Rb (Ser807/811)	Cell Signaling	Cat# 9308; RRID:AB_331472
Rabbit anti-Topoisomerase IIα	Cell Signaling	Cat# 12286; RRID:AB_2797871
Rabbit anti-*C9orf72*	Cell Signaling	Cat# 64196; RRID:AB_2833080
Rabbit anti-PUMA (BBC3)	Cell Signaling	Cat# 12450; RRID:AB_2797920
Rabbit anti-PSD95	Cell Signaling	Cat# 3450S; RRID:AB_2292883
Rabbit anti-Synapsin-1	Cell Signaling	Cat# 5297S; RRID:AB_2616578
Goat anti-Choline Acetyltransferase (ChAT)	Millipore	Cat# AB144P; RRID:AB_2079751
Mouse anti-β-Actin	Cell Signaling	Cat# 3700; RRID:AB_2242334
Mouse anti-β-Actin	AB clonal	Cat# AC004; RRID:AB_2737399
Mouse anti-GAPDH	AB clonal	Cat# AC002; RRID:AB_2736879
Goat anti-rabbit IgG(H + L) biotinylated	Vector laboratories	Cat# BA-1000; RRID:AB_2313606
Goat anti-mouse IgG(H + L) biotinylated	Vector laboratories	Cat# BA-9200; RRID:AB_2336171
IRDye® 800CW Streptavidin Secondary Antibody	LI-COR Biosciences	Cat# 926-32230
IRDye® 680RD Streptavidin Secondary Antibody	LI-COR Biosciences	Cat# 926-68079
IRDye® 800CW Donkey anti-Mouse IgG Secondary Antibody	LI-COR Biosciences	Cat# 926-32212; RRID:AB_621847
IRDye® 680LT Goat anti-Rabbit IgG Secondary Antibody	LI-COR Biosciences	Cat# 926-68021; RRID:AB_10706309
Donkey anti-rabbit IgG, Alexa Fluor 568	Thermo Fisher Scientific	Cat# A10042; RRID:AB_2534017
Donkey anti-mouse IgG, Alexa Fluor 488	Thermo Fisher Scientific	Cat# A21202; RRID:AB_141607
Donkey anti-goat IgG, Alexa Fluor 647	Thermo Fisher Scientific	Cat# A21447; RRID:AB_2535864

**Chemicals, peptides, and recombinant proteins**		

Matrigel	Corning	Cat# 354277
mTeSR Plus medium	STEMCELL Technologies	Cat# 100-0276
Neurobasal medium	Gibco	Cat# 21103049
DMEM/F12	Gibco	Cat# 21331020
N2 Supplement	Gibco	Cat# A13707-01
B27 Supplement	Gibco	Cat# 17504001
GlutaMAX	Gibco	Cat# 35050061
Ascorbic acid	Sigma-Aldrich	Cat# A4403
CHIR99021	STEMCELL Technologies	Cat# 72052
SB431542	STEMCELL Technologies	Cat# 72234
DMH1	STEMCELL Technologies	Cat# 73634
Retinoic acid	Sigma-Aldrich	Cat# R2625
Purmorphamine	STEMCELL Technologies	Cat# 100-1049
BDNF	PeproTech	Cat# 450-02
GDNF	PeproTech	Cat# 450-10
cAMP	Biogems	Cat# 1698950
Compound E	STEMCELL Technologies	Cat# 73954
Accutase	Gibco	Cat# A1110501
Cas9 recombinant protein	Integrated DNA Technologies	Cat# 1081066
Propidium Iodide	Invitrogen™	Cat# P43566
RNase A (DNase-free)	Thermo Scientific	Cat# EN0531
Poly(GR), Poly(PR), Poly(GP) peptides	Custom synthesized	N/A
Palbociclib	Pfizer	Cat# 0332991
Halt Protease and Phosphatase Inhibitor Cocktail	Thermo Scientific	Cat# 78440
RIPA Buffer	Thermo Scientific	Cat# 89901
Hochest	Thermo Fisher Scientific	Cat# 33342
DMSO	Sigma	Cat# D8418
16% paraformaldehyde	Electron Microscopy Sciences	Cat# 15710
NuPAGE MOPS SDS 20X running buffer	Invitrogen	Cat# NP0001
NuPAGE 20X transfer buffer	Invitrogen	Cat# NP00061
Intercept® Blocking Buffer	LI-COR	Cat# 927-60001
10% Tween 20	Bio-Rad	Cat# 1610781
Triton X-100	Bio-Rad	Cat# 1610407

**Critical commercial assays**		

PureLink RNA Mini Kit	Invitrogen	Cat# 12183018A
iScript cDNA Synthesis Kit	Bio-Rad	Cat# 1708891
SYBR Green PCR Master Mix	Applied Biosystems	Cat# 4309155
ApopTag Fluorescein *In Situ* Apoptosis Detection Kit	Millipore	Cat# S7110

**Deposited data**		

Raw and processed data files for single-cell RNA-seq	This paper	GSE219281

**Experimental models: Cell lines**		

Control iPSC lines (35L5, 35L11, 37L20)	(Lopez-Gonzalez et al.,^6^)	N/A
*C9orf72* iPSC lines (16L14, 40L3, 42L11)	(Lopez-Gonzalez et al.,^6^)	N/A

**Oligonucleotides**		

gRNA sequences 5′GCTTACTGGGACAATATTC TTGG	Integrated DNA Technologies	N/A
gRNA sequences 3′CCTTCGAAATGCAGAGAGTGGTG	Integrated DNA Technologies	N/A

**Software and algorithms**		

GraphPad Prism 9.1	GraphPad Software	https://www.graphpad.com/
Image Studio	LI- COR Biosciences	https://www.licorbio.com/image-studio
FlowJo (v10.8.2)	FlowJo software	https://www.flowjo.com/flowjo10/download
BD FACSDiva software	BD Biosciences	https://www.bdbiosciences.com/en-us
R software (v4.4.1)	R Foundation for Statistical Computing	https://www.r-project.org/
Seurat (R toolkit for single cell genomics) (v5.2.1)	N/A	https://bioconductor.org/packages/release/BiocViews.html#___Software
inferCNV (v1.20.0)	N/A	https://anaconda.org/bioconda/bioconductor-infercnv/files
Custom code	This paper	https://doi.org/10.5281/zenodo.15593963

**Other**		

Multiskan SkyHigh Microplate Spectrophotometer	Thermo Scientific	N/A
C1000 Touch™ Thermal Cycler	Bio-Rad	N/A
QuantStudio™6 Flex Real-Time PCR System	Thermo Scientific	N/A
Odyssey® DLx Imaging System	LI- COR Biosciences	N/A
BD LSRFortessa™ Cell Analyzer	BD Biosciences	N/A

### Experimental model and study participant details

#### Human iPSC lines

In this study we used motor neurons differentiated from three control iPSC lines: 35L5, 35L11 and 37L20; three *C9orf72* carriers derived iPSC lines: 16L14, 40L3 and 42L11, these iPSC lines have been thoroughly characterized and published previously^6^^,^^30^^,^^31^ and are described in (Table S1).

### Method details

#### Motor neuron differentiation

Motor neurons were differentiated using previously published methods.^11^ Briefly, iPSCs were grown in Matrigel-coated wells using mTeSR plus medium (Stem Cell Technologies). Then medium was replaced with neuroepithelial progenitor (NEP) medium, neurobasal (Gibco), DMEM/F12 (Gibco) medium at 1:1, 0.5X N2 (Gibco), 0.5X B27(Gibco), 1X Glutamax (Invitrogen), 0.1 mM ascorbic acid (Sigma), 3 μM CHIR99021 (StemCell technologies), 2 μM SB431542 (StemCell technologies) and 2 μM DMH1 (Tocris Bioscience). Media has replaced with fresh NEP media every other day for 6 days, NEPs were dissociated with accutase and replated into Matrigel-coated wells, with motor neuron progenitor induction medium (NEP medium with 0.1 μM retinoic acid and 0.5 μM purmorphamine. Then we used MNP media every other day and cells were grown for 6 days. MNPs were then dissociated to generate suspension cultures of neurosphere and grown for 6 days. Neurospheres were dissociated into single cells and plated on Matrigel-coated plates or coverslips in motor neuron medium composed of Neurobasal medium, 1X B27, 1X Glutamax, 10 ng/mL BDNF (PeproTech), 10 ng/mL GDNF (PeproTech), 0.5 μM cAMP (Biogems) and 0.1 μM Compound E (Calbiochem). Post-mitotic motor neurons were cultured up to two months and analyzed at different time points.

#### Generation of heterozygous and homozygous *C9orf72* iPSC lines

Human control iPSC line 35L11 was nucleofected with recombinant Cas9 protein (Integrated DNA technologies) with the following synthetic gRNA sequences 5′ GCTTACTGGGACAATATTC TTGG and 3′ CCTTCGAAATGCAGAGAGTGGTG. Then iPSC lines were clonally isolated and characterized to measure *C9orf72* protein levels and pluripotency markers.

#### RNA extraction and quantitative real-time PCR

Total RNA was extracted from motor neurons at different stages or after specific treatment using PureLink RNA Mini Kits (Invitrogen) as per manufacturer’s instructions. RNA concentration and purity were measured by Multiskan SkyHigh Microplate Spectrophotometer (Thermo Scientific) and reverse transcribed to synthesize cDNA by iScript cDNA Synthesis Kit (Biorad) according to manufacturer’s instructions using C1000 Touch Thermal Cycler (Biorad). cDNA (10 ng) was used for quantitative real-time PCR by SYBR Green PCR Master Mix (Applied Biosystem) in a QuantStudio6 Flex Real-Time PCR System using the primers listed in (Table S2). Ct values for each gene were normalized to that of housekeeping gene, GAPDH. Relative mRNA expression was analyzed by the 2 delta-delta Ct method.

#### Western blot analysis

Motor neuron cultures were lysed with Pierce RIPA Buffer (Thermo Scientific) supplemented with Halt Protease and Phosphatase Inhibitor Cocktail (Thermo Scientific). Protein lysates were analyzed by SDS-PAGE followed by immunoblotting to detect specific protein expression. Primary antibodies and their concentration used were listed in (Table S3). Membranes were incubated overnight with diluted primary antibody at 4°C on orbital shaker and then washed with TBS-T to remove the excess primary antibody. Membranes were incubated with appropriate anti-mouse or antirabbit IR-Dye secondary antibodies (LI-COR Biosciences) and incubated for 1h at room temperature and then imaged with an Odyssey DLx Imaging System (LI- COR Biosciences). Immunoblotting images were analyzed by Image Studio (LI-COR Biosciences) and relative expression was measured by assessing β-actin levels as loading controls.

#### Flow cytometry

Cell cycle progression in iPSC-derived motor neurons from controls and *C9orf72* and C9orf72 treated with palbociclib were analyzed by Flow cytometry. 2-month-old-motor neurons were washed with ice-cold PBS harvested and then fixed with 70% ethanol overnight at 4°C. Ethanol was removed from the fixed cells and the cells were washed with PBS. Then neurons were resuspended in 1 mL of Propidium Iodide (PI) staining solution (20 μg/mL PI, 200 μg/mL DNase-free RNaseA and 0.1% Triton X-100 FBS in PBS).10,000 events per condition were run using BD LSRFortessa™ Cell Analyzer (BD Biosciences) and PI fluorescence was recorded by BD FACSDiva software (BD Biosciences). Data were derived without gating strategy applied. PI (+) events were then gated for singlets and plotted on a histogram using FlowJo software (v10.8.2).

#### DRPs and palbociclib treatment

1-month-old control iPSC-derived neurons were treated with synthetic DRPs, poly-GR, poly-PR or poly-GP, using 1 and 2 μM concentrations. After 48 h dipeptide repeat protein containing media were removed and cells were rinsed with fresh neurons culture media. 1-month-old iPSC-derived neurons from *C9orf72* were used for palbociclib treatment, we used two concentrations 1 and 5 μM or DMSO (vehicle control). Neurons were then cultured for 1 month in presence of palbociclib, collected and pelleted for either RNA or protein extraction or fixed using the above-mentioned procedure using ethanol and subjected to cell cycle analysis by Flow cytometry.

#### TUNEL assay

We performed TUNEL assay in 2-month-old iPSC-derived neurons, neurons were fixed with 4% paraformaldehyde to perform TUNEL assay with the ApopTag® Fluorescein *in Situ* Apoptosis Detection Kit (Millipore). Afterwards, we performed TUNEL assay immunostaining with the primary antibody goat anti ChAT (Table S2).

#### SnRNA-seq analysis

Publicly available single-nucleus RNA sequencing (snRNA-seq) datasets were retrieved from the Gene Expression Omnibus (GEO) under accession number GSE219281. The datasets included postmortem human brain samples from neurologically healthy controls and amyotrophic lateral sclerosis (ALS) patients harboring *C9orf72* hexanucleotide repeat expansions. All data processing and analysis were conducted using R (v4.4.1). Raw count matrices and sample metadata were downloaded and processed using the Seurat package (v5.2.1). Data were normalized using the SCTransform method, and highly variable genes were identified for downstream analyses. Dimensionality reduction was performed using principal component analysis (PCA), and nuclei were clustered using the Leiden algorithm. Two-dimensional visualization of the clustered nuclei was performed using Uniform Manifold Approximation and Projection (UMAP). Cell type annotation was conducted by comparing cluster-specific marker genes with canonical cell-type markers curated from published literature. Cell cycle phase scores were calculated using the AddModuleScore function from the Seurat package, with previously reported cell cycle-related genes, and violin plots were generated to visualize cell cycle distributions across control and patient groups. To assess large-scale genomic instability, inferCNV (v1.20.0) was applied to the processed expression matrix, using normal cells from control individuals as a reference population. Copy number variation (CNV) scores and heatmaps were generated for visualization. Genes located in regions with predicted CNV gains or losses were subjected to pathway enrichment analysis using Enrichr, focusing on Gene Ontology Biological Process (GO-BP) terms. Scatter plots were generated to present significantly enriched pathways related to cell cycle regulation.

### Quantification and statistical analysis

Statistical analyses were performed in GraphPad Prism version 9.1 (La Jolla, CA).

Differences between two means were analyzed by two-tailed t-tests with Welch’s correction. One-way ANOVA followed by Tukey’s multiple-comparison test were used to analyzed significant differences among multiple means.
