# Supplementary material for: Aberrant CDK4/6-driven cell-cycle reentry drives neuronal loss and defines a therapeutic target in C9orf72 ALS/FTD
Source: iScience. 2026 Jan 2;29(2):114596. doi: 10.1016/j.isci.2025.114596 (PMC12856326; doi:10.1016/j.isci.2025.114596)
Supplement: Document S1. Figures S1–S4 and Tables S1–S3 [file mmc1.pdf]

iScience, Volume *29*

Supplemental information

Aberrant CDK4/6-driven cell-cycle reentry drives neuronal loss and defines a therapeutic target in C9orf72 ALS/FTD

Ling Lian, Hayley Robinson, Noah Daniels, G. Aleph Prieto, Gunnar H.D. Poplawski, and Rodrigo Lopez-Gonzalez

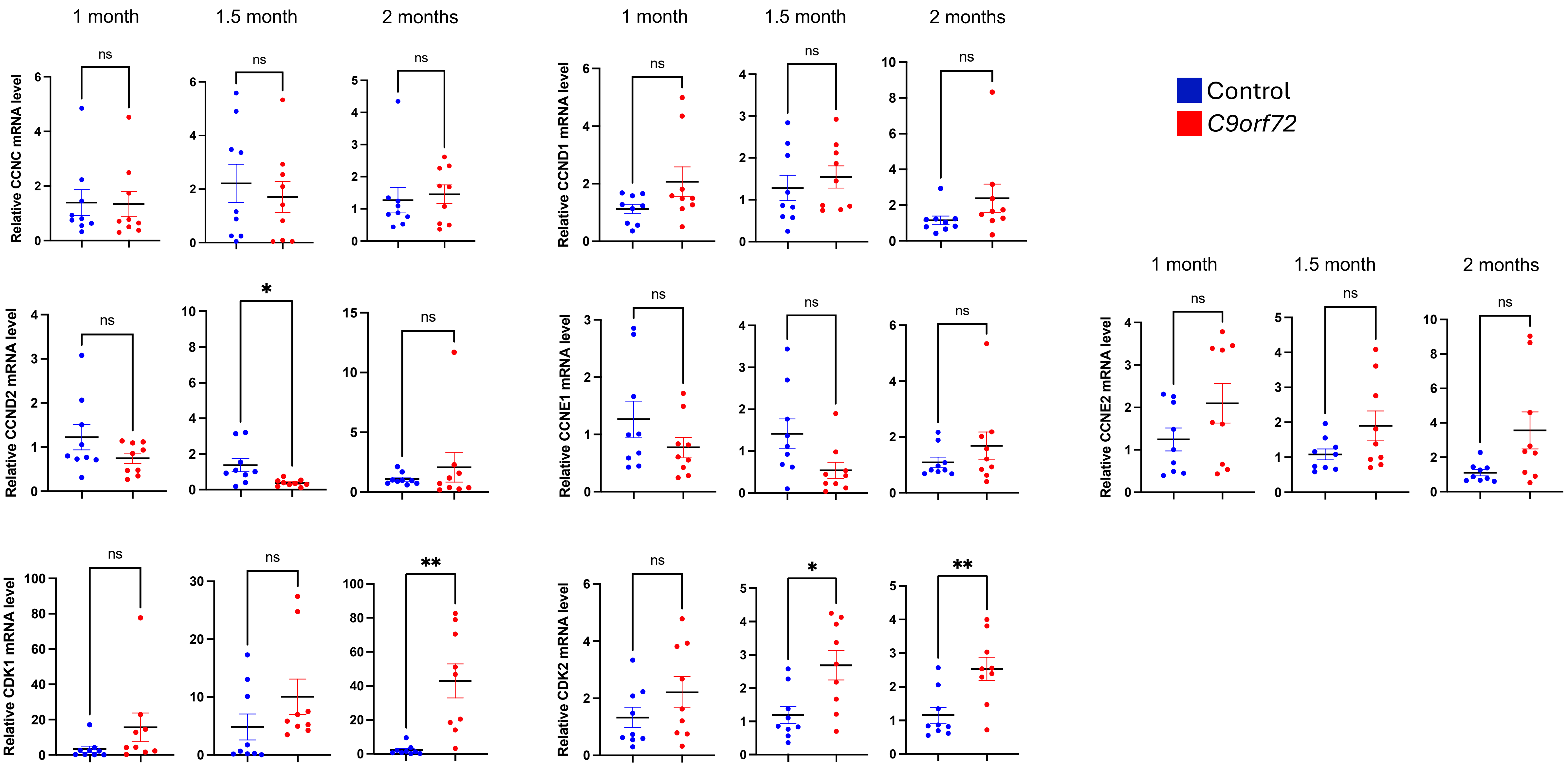

Supplementary Figure 1. Expression of cyclins and CDKs in *C9orf72* iPSC-derived neurons. Relative mRNA expression of cell cycle regulation genes CCNC, CCND1, CCND2, CCNE1, CCNE2, CDK1, CDK2 and in iPSC derived *C9orf72* motor neurons compared to control motor neurons. Data are presented as mean ± SEM.

Data presented is from 3 control iPSC and 3 C9orf72 iPSC lines from 3 independent differentiation experiments. Two-tailed t-test with Welch's correction was applied. ns, not significant, *p<0.05, **p<0.01.

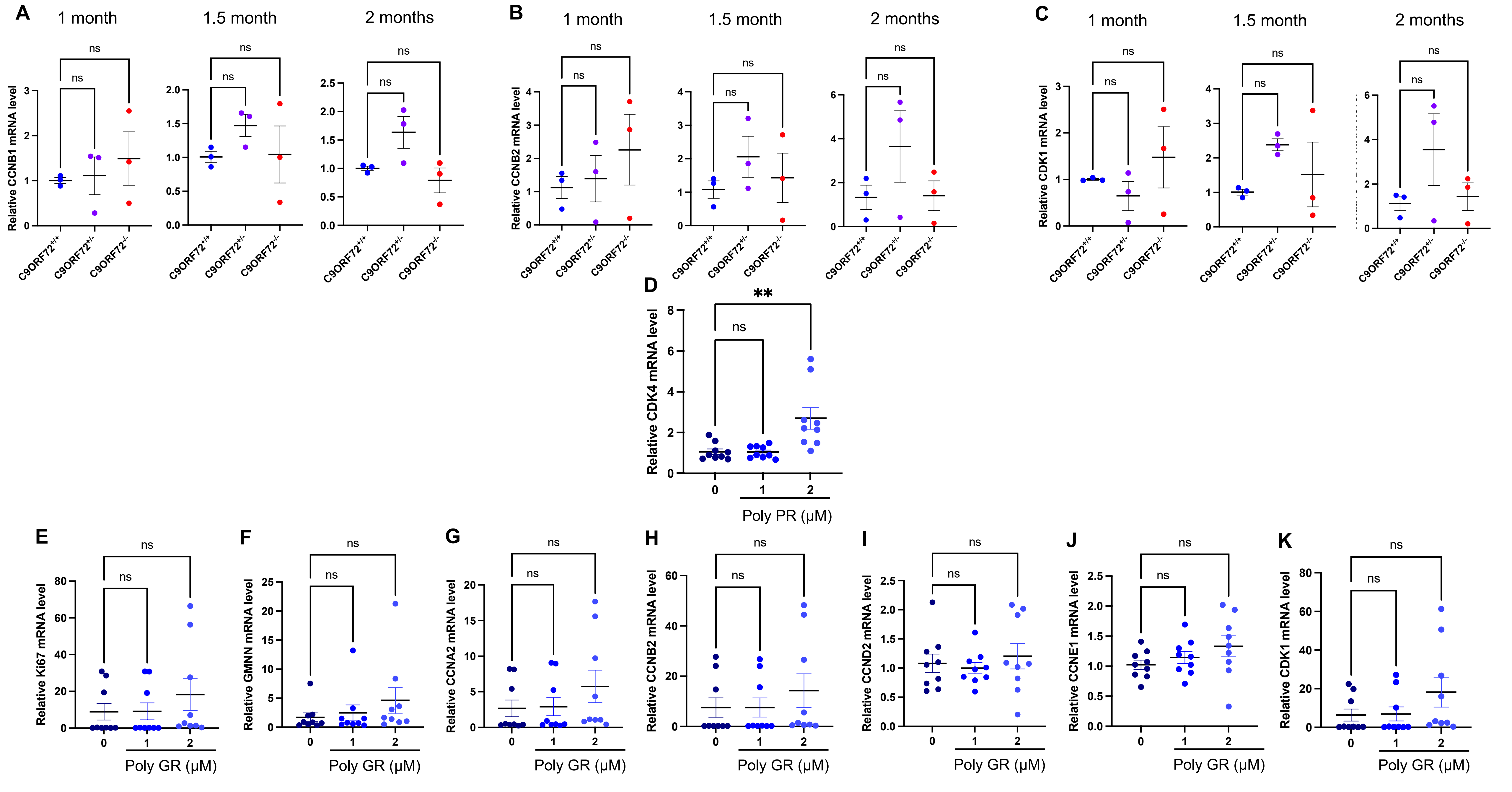

Supplementary Figure 2. Cyclin and CDK expression in heterozigous and homozigous knockout C9orf72 neurons and iPSC-derived neurons treated with DPRs (A - C) Relative mRNA expression of CCNB1, CCNB2 and CDK1 in motor neurons differentiated from *C9orf72* homozygous and heterozygous knock out lines compared to their parental line. (D) mRNA levels of CDK4 in control iPSC-derived motor neurons treated with poly (PR) (1 and 2 µM). (E-K) mRNA levels of Ki67, GMNN, CCNA2, CCNB2, CCND2, CCNE1 and CDK1 in control iPSC-derived motor neurons treated with poly (GR) (1 and 2 µM).
Data are presented as mean ± SEM.
Data presented in (A-C) is from 3 independent differentiation experiments of control parental iPSC lines and *C9orf72* heterozygous and homozygous Knockout lines. Two-tailed t-test with Welch's correction was applied. ns, not significant. Data in (D-K) is from 3 control iPSC lines from 3 independent differentiation experiments. [illegible]

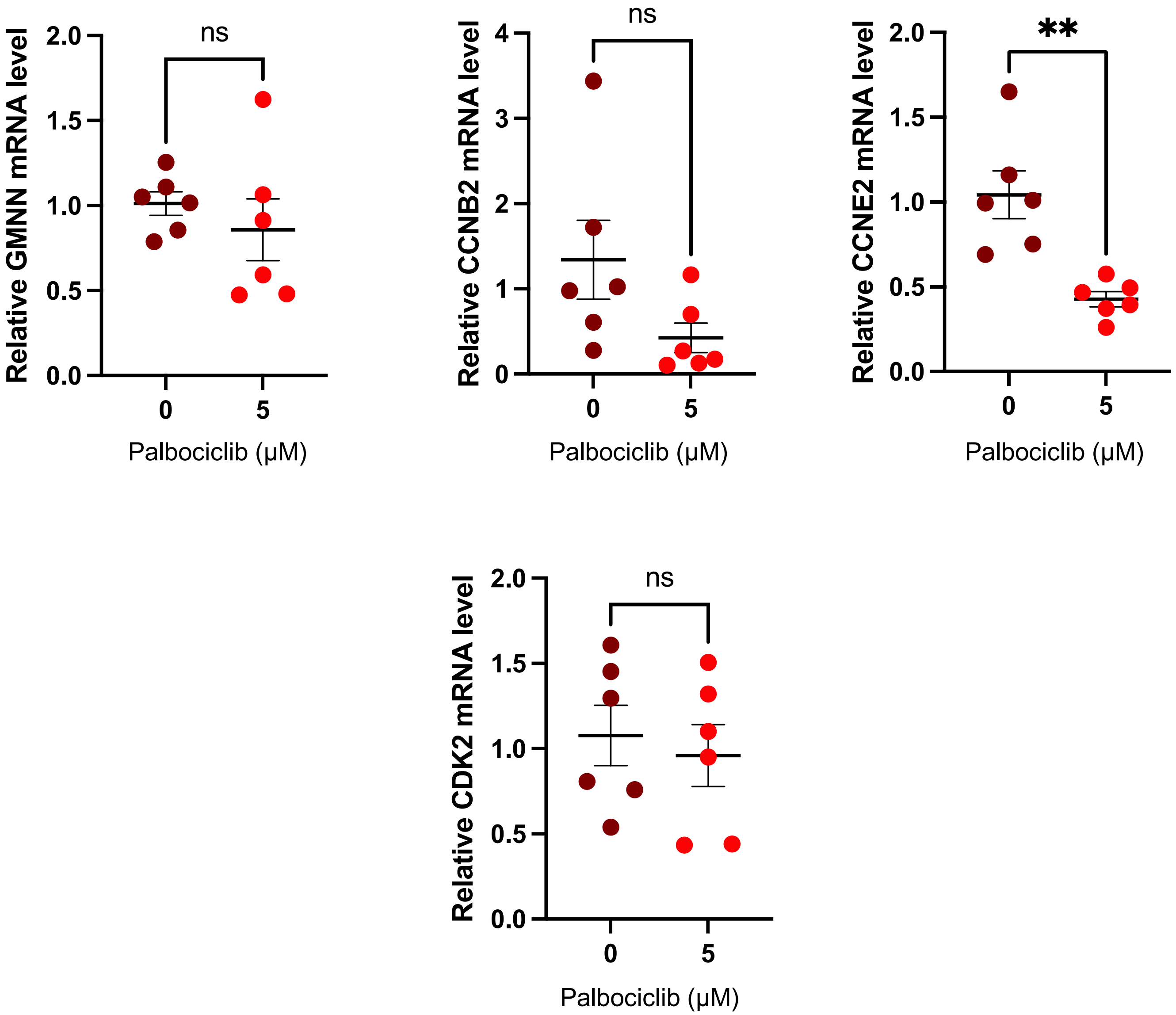

Supplementary Figure 3. Cell cycle marker expression in Palbociclib-treated iPSC-derived neurons from *C9orf72* carriers. Relative mRNA levels of GMNN, CCNB2, CCNE2, and CDK2 in 2-month-old *C9orf72* iPSC-derived motor neurons treated with Palbociclib 1 and 5 μM.
Data are presented as mean ± SEM.
Data is from 2 *C9orf72* iPSC lines from 3 independent experiments. Two-tailed t-test with Welch's correction was applied **$p<0.01$.

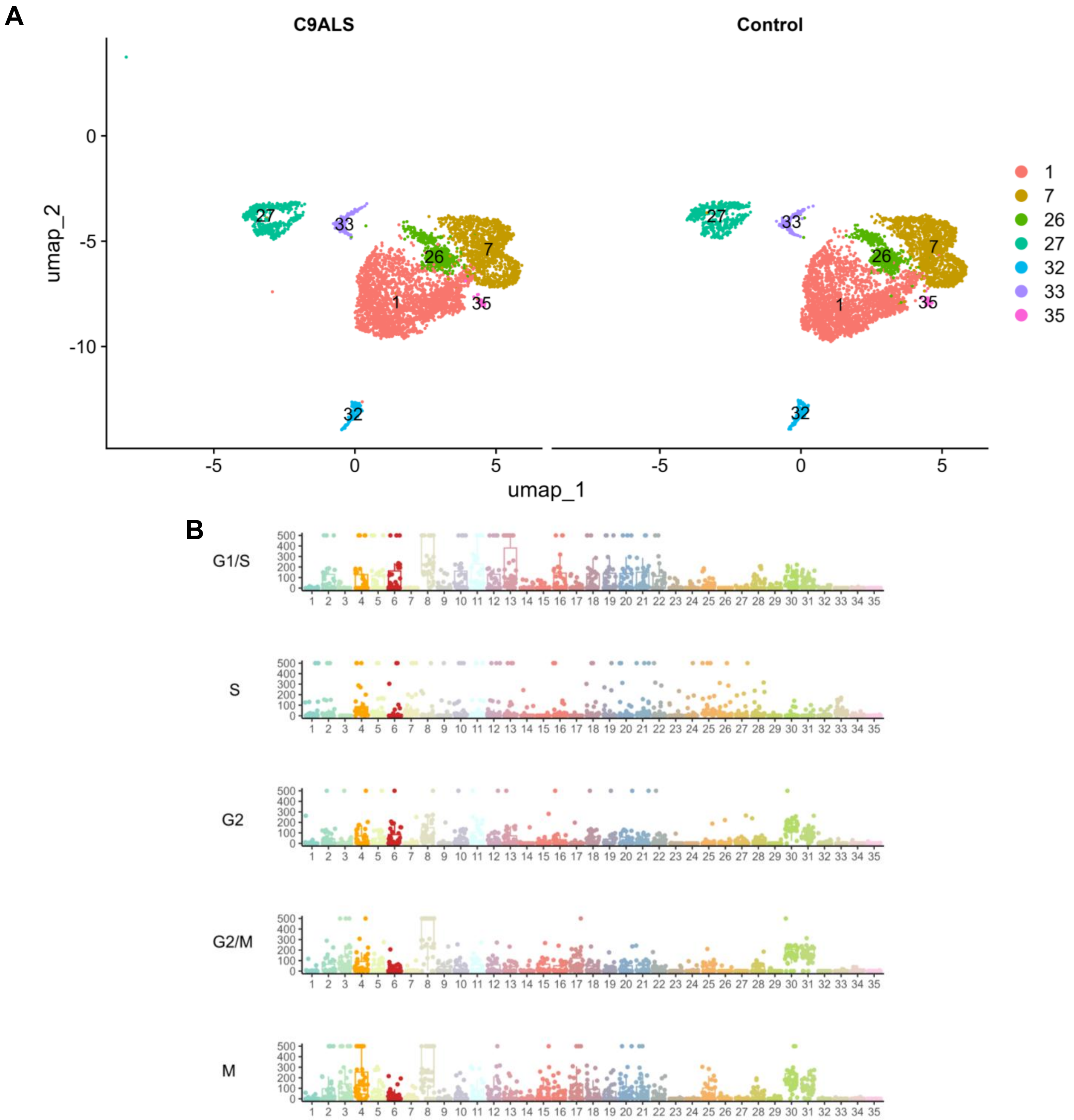

Supplementary Figure 4. Cell cycle dysregulation in excitatory neuron subclusters from *C9orf72* carriers (A) UMAP plot of single-nucleus RNA sequencing data from excitatory neurons C9orf72 ALS and control groups. (B) The density and distribution of -log10(p-values) (y-axis) within each subcluster illustrates the variability and statistical significance of cell cycle scores relative to other subclusters.

Supplementary Table 1. Summary of iPSC lines used in this study

iPSC line name	Source	Age	Clinical Diagnosis	Number of expanded repeats	Gender
**Control**					
35L5	Skin fibroblasts	56	Healthy control	N/A	Male
35L11	Skin fibroblasts	56	Healthy control	N/A	Male
37L20	Skin fibroblasts	65	Healthy control	N/A	Female
C9orf72					
16L14	Skin fibroblasts	50	FTD	~590	Male
40L3	Skin fibroblasts	65	FTD	~900	Male
42L11	Skin fibroblasts	59	FTD	~1000	Female

Abbreviations:
FTD: Frontotemporal dementia

Supplementary Table 2. List of primers used in this study

Targeted genes	Forward primers (5' to 3')	Reverse primer (5' to 3')
Ki67	CTTTGGGTGCGACTTGACGA	ACAACTCTTCCACTGGGACG
GMNN	GTCAGTTGGTCACGTGGTTG	TGAAGCACAGAAGATGGGTGG
CCNA2	CTCTACACAGTCACGGGACAAAG	CTGTGGTGCTTTGAGGTAGGTC
CCNB1	GACCTGTGTCAGGCTTTCTCTG	GGTATTTTGGTCTGACTGCTTGC
CCNB2	TGGAAAAGTTGGCTCCAAAG	TCAGAAAAAGCTTGGCAGAGA
CCNC	GCAGAAAGATGCCAGGCAATGG	CTCTCATCGAAATTCTTCCACTGC
CCND1	GCTGTGCATCTACACCGACA	TTGAGCTTGTTCACCAGGAG
CCND2	GGACATCCAACCCTACATGC	CGCACTTCTGTTCCTCACAG
CCNE1	GGCCAAAATCGACAGGAC	GGGTCTGCACAGACTGCAT
CCNE2	GCCATTGATTCATTAGAGTTCCA	CTGTCCCACTCCAAACCTG
CDK1	GGAAACCAGGAAGCCTAGCATC	GGATGATTCAGTGCCATTTTGCC
CDK2	ATGGATGCCTCTGCTCTCACTG	CCCGATGAGAATGGCAGAAAGC
CDK4	CCATCAGCACAGTTCGTGAGGT	TCAGTTCGGGATGTGGCACAGA
CDK6	GGATAAAGTTCCAGAGCCTGGAG	GCGATGCACTACTCGGTGTGAA
P19	GTGCATCCCGACGCCCTCAAC	TGGCACCTTGCTTCAGCAGCTC
P21	AGTCAGTTCCTTGTGGAGCC	GACATGGCGCCTCCTCTG
TOP2α	AGTGTCACCATTGCAGCCTGT	CAATGTAGGTGTCTGGGCGG
GAPDH	TGCACCACCAACTGCTTAGC	GGCATGGACTGTGGTCATGAG

Supplementary Table 3. Antibodies used in this study

Antibody	Vendor	Catalog No.	Source	Dilution used
CCNA2	Cell Signaling	67955	Rabbit	1:1000
C9orf72	Cell Signaling	12231	Rabbit	1:1000
CDK4	Cell Signaling	12790	Rabbit	1:1000
Topoisomerase IIα	Cell Signaling	12286	Rabbit	1:1000
Phospho-RB (S780)	Cell Signaling	9307	Rabbit	1:1000
β-Actin	Abclonal	AC004	Mouse	1:10,000
β-Actin	Abclonal	AC038	Rabbit	1:10,000
